# Olfactory organ of *Octopus vulgaris*: morphology, plasticity, turnover and sensory characterization

**DOI:** 10.1242/bio.017764

**Published:** 2016-04-11

**Authors:** Gianluca Polese, Carla Bertapelle, Anna Di Cosmo

**Affiliations:** Department of Biology, University of Napoli Federico II, Napoli, NA 80126, Italy

**Keywords:** *Octopus vulgaris*, Olfactory organ, Olfactory sensory neurons, Olfactory marker protein, PCNA

## Abstract

The cephalopod olfactory organ was described for the first time in 1844 by von Kölliker, who was attracted to the pair of small pits of ciliated cells on each side of the head, below the eyes close to the mantle edge, in both octopuses and squids. Several functional studies have been conducted on decapods but very little is known about octopods. The morphology of the octopus olfactory system has been studied, but only to a limited extent on post-hatching specimens, and the only paper on adult octopus gives a minimal description of the olfactory organ. Here, we describe the detailed morphology of young male and female *Octopus vulgaris* olfactory epithelium, and using a combination of classical morphology and 3D reconstruction techniques, we propose a new classification for *O. vulgaris* olfactory sensory neurons. Furthermore, using specific markers such as olfactory marker protein (OMP) and proliferating cell nuclear antigen (PCNA) we have been able to identify and differentially localize both mature olfactory sensory neurons and olfactory sensory neurons involved in epithelium turnover. Taken together, our data suggest that the *O. vulgaris* olfactory organ is extremely plastic, capable of changing its shape and also proliferating its cells in older specimens.

## Abstract (precis)

**Summary:** A 3D reconstruction of the *Octopus vulgaris* olfactory organ, as well as the localization of PCNA and (for the first time) OMP-like proteins in cephalopod mature olfactory chemosensory neurons are described.

## INTRODUCTION

Cephalopods are considered ‘advanced invertebrates’ for many reasons, particularly the size of their brain that represents a conspicuous fraction of their body mass (Packard, 1972). They have evolved a complex nervous system (Nixon and Young, 2003) while maintaining the basal molluscan plan of tetraneury (Wanninger, 2009; Moroz, 2009). Their brain is encapsulated in a cartilaginous cranium and lies between the eyes. The supra- and sub- esophageal masses show a multi-lobed organization and lie between two large optic lobes. This complex structural organization functions hierarchically: motoneurons of the lower and intermediate motor centers, situated for the most part in the suboesophageal mass, innervate effectors. These centers are controlled by the higher motor centers, by neurons of the basal lobes which in turn are controlled by the optic lobes (Boycott, 1961; Young, 1971). This complex nervous system allows cephalopods to display discriminative (Cole and Adamo, 2005), observational (Suboski et al., 1993), associative learning (Agin et al., 2006a), and imprinting (Darmaillacq et al., 2006). These learning abilities are associated with long-term memory (Sanders, 1975; Agin et al., 2006b; De Lisa et al., 2012a,b) and spatial memory (Alves et al., 2007).

The sense organs of cephalopods are the most sophisticated of all the invertebrates (Packard, 1972; Young, 1977, 1989; Budelmann, 1995, 1996; Messenger, 1977; Anderson et al., 2010). Cephalopods have been known for well developed eyes and complex visual behavior (Hanlon and Messenger, 1996; Grable et al., 2002; Zylinski et al., 2009; Yoshida et al., 2015), for highly developed vestibular system, a ‘lateral line analogue’, and for a primitive ‘hearing’ system (Bleckmann et al., 1991; Budelmann and Williamson, 1994; King et al., 2003; Williamson and Chrachri, 2007).

Cephalopods possess chemoreceptors in the epidermis (see Budelmann, 1996) including numerous isolated sensory neurons all over the body surface (Graziadei, 1964; Sundermann-Meister, 1978; Boletzky, 1989; Fioroni, 1990; Mackie, 2008; Baratte and Bonnaud, 2009) and mostly in the hundreds of suckers of octopods as well as, but in less concentration, on squid and cuttlefish suckers, lips and mouth (Wells et al., 1965; Wells, 1978; Boyle, 1983; Anraku et al., 2005).

In coleoid cephalopods a small pit of ciliated cells is present on either side of the head below the eyes close to the mantle edge. These structures represent the olfactory organs as recognized by von Kölliker (1844) and Watkinson (1909). While several functional studies have been conducted on decapods, demonstrating their role in mate choice of squid and cuttlefish and the improvement of predation on crabs by cuttlefish (Boyle, 1983, 1986; Chase and Wells, 1986; Lee, 1992; Lucero et al., 1992, 1995, 2000; Budelmann et al., 1997; Boal and Golden, 1999; Piper and Lucero, 1999; Mobley et al., 2007, 2008a,b; Villanueva and Norman, 2008), very little is known about octopods (Walderon et al., 2011). The *Octopus vulgaris* olfactory organ has the typical morphology of a chemoreceptor structure (Woodhams and Messenger, 1974; Wildenburg, 1995, 1997) resulting in a ciliated epithelium lying in a pair of pits, one on each side of the head, as in other cephalopods and it has been considered the homologue of Nautilus rhinophore, a specialized short hollow tentacle lodged below the eye (Young, 1965; Basil et al., 2000; Ruth et al., 2002; Jereb and Roper, 2005).

From the olfactory pit nerve fibers arise and form a defined olfactory nerve, which crosses the floor of the orbit and enters the olfactory lobe.

This lobe, situated on the optic tract, close to optic gland, organized in three interconnected lobules, receives fibers also from dorsal basal and optics lobes and sends fibers to the basal and subpedunculate lobes (Messenger, 1967). For these neuroanatomical connections it constitutes a center of convergence and interception of fibers coming from lobes involved in the control of motor program and reproduction. (Di Cosmo and Di Cristo, 1998; De Lisa et al., 2012a,b; Di Cosmo and Polese, 2013, 2014; Di Cristo, 2013; Polese et al., 2015).

Our group demonstrated in *O. vulgaris* functional differences among the lobules of the olfactory lobe based on differential distributions of peptidergic neurons in these brain regions (Di Cosmo and Di Cristo, 1998; Di Cosmo and Polese, 2013, 2014). Recently Polese et al. (2015) discovered the presence of APGWamide, FMRFamide, NPY and GnRH in the olfactory sensory neurons (OSNs) and fibers of the *O. vulgaris* olfactory organ proposing a new model of control of reproduction based on chemical cues.

Cephalopods, as suggested by several studies, are able to detect chemical cues either through contact or distant chemoreception (Boyle, 1983, 1986; Chase and Wells, 1986; Lee, 1992; Boal and Golden, 1999; Alves et al., 2007). The behavioral evidence for distant chemoreception show that the addiction of fish juice to the water causes, in octopus (Wells, 1963) and cuttlefish (Messenger, 1977), active movements. In this context, cephalopods produce the ink that they use as direct deterrent of predators and as an alarm cue for conspecifics (Palumbo et al., 1999; Di Cosmo, 2003; Di Cosmo et al., 2006; Derby, 2014).

Boal (1997) argued that female mate choice in cuttlefish was more likely to be based on olfactory cues rather than visual cues. Adding dilute extracts of crabs to the water supply increased the ventilation rate of octopus (Boyle, 1983) and typical signs of alarm are shown by octopus when exposed to seawater in which a moray eel had been living (Mac Ginitie and Mac Ginitie, 1968). Furthermore the ability to detect the sex of conspecifics at a distance, in octopuses, could facilitate reproduction and also problem solving ability (Boal, 2006; Anderson et al., 2010). Nevertheless a blinded octopus will move towards a scent it perceives as a food source (Chase and Wells, 1986). Recently Walderon et al. (2011) demonstrated that octopuses respond to chemical signals from conspecifics and detect a wide range of odors as food or non-food (seaweed). However as most coleoids are nocturnal or live at depths where little light is present, the ability to track prey, partner and predator by scent is crucial to their success (Joll, 1977; Budelmann, 1996). This strongly suggests that the coleoid cephalopods, octopods, cuttlefishes and squids use distance chemoreception and the ability to integrate chemical signals with the stimuli perceived by other their sense organs allowing them to shape their sophisticated behavior in the sea.

To date the morphology, the plasticity, the proliferation capability of olfactory sensory neurons (OSNs), and the sensory characterization of the *O. vulgaris* olfactory organ remain to be elucidated.

In order to shed more light on these crucial features here we provide: a detailed description of olfactory epithelium (OE) of young male and female *O. vulgaris*; three-dimensional reconstruction of the OE; the localization of proliferating cell nuclear antigen (PCNA) as a molecular marker of cell cycle progression and DNA replication; the first time localization of olfactory marker protein-like (OMP) in the *O. vulgaris* olfactory epithelium as a marker of mature olfactory chemosensory neurons.

## RESULTS

### Overview of olfactory organ morphology

**Fig. 1.**
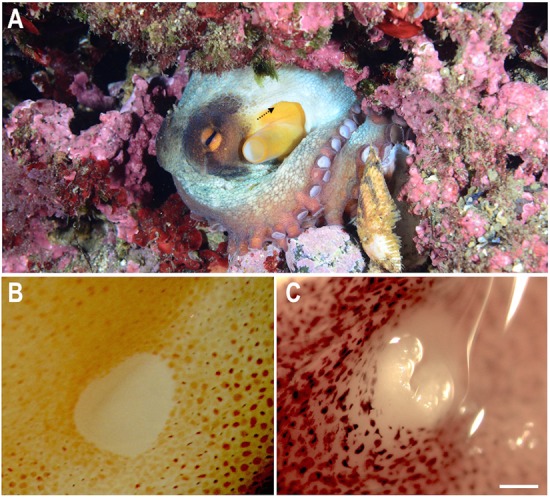
**Overview of *O. vulgaris* olfactory organ.** (A) Entry to mantle cavity of a young *O. vulgaris* in its natural environment: the arrow indicates the position of the olfactory organ and the direction of water flow. (B) Magnification of the olfactory organ in relaxed position. (C) Magnification of the erect olfactory organ when extended out of the olfactory pit. Scale bar=1000 µm.

The paired olfactory organs of *O. vulgaris* are localized on each side of the head at the inhalant entrance to the mantle cavity. They are hidden in skin folds and appear to be small white patches when the skin is stretched, due to the absence of chromatophores (Fig. 1). The olfactory organs of 12 young octopuses were used in this study because they afforded better visualization of the organs respect to larger animals. Young olfactory organs were not yet fully covered and enclosed in the skin folds. With further growth and development, the olfactory organs become hidden in the skin folds.

### Histology and cell types

The olfactory organs are composed of sustentacular and epithelial sensory cells. The surface of the OE is organized in a pseudo-stratified, columnar and ciliated epithelium and bulges into the olfactory pit to form the olfactory protuberance (OP). Each OE appear capable of erection to expose the sensory epithelium (see below).

The surface layer is characterized by different types of OSNs and sustentacular cells, below which a multilayer of a ring shaped OSNs are arranged.

**Fig. 2.**
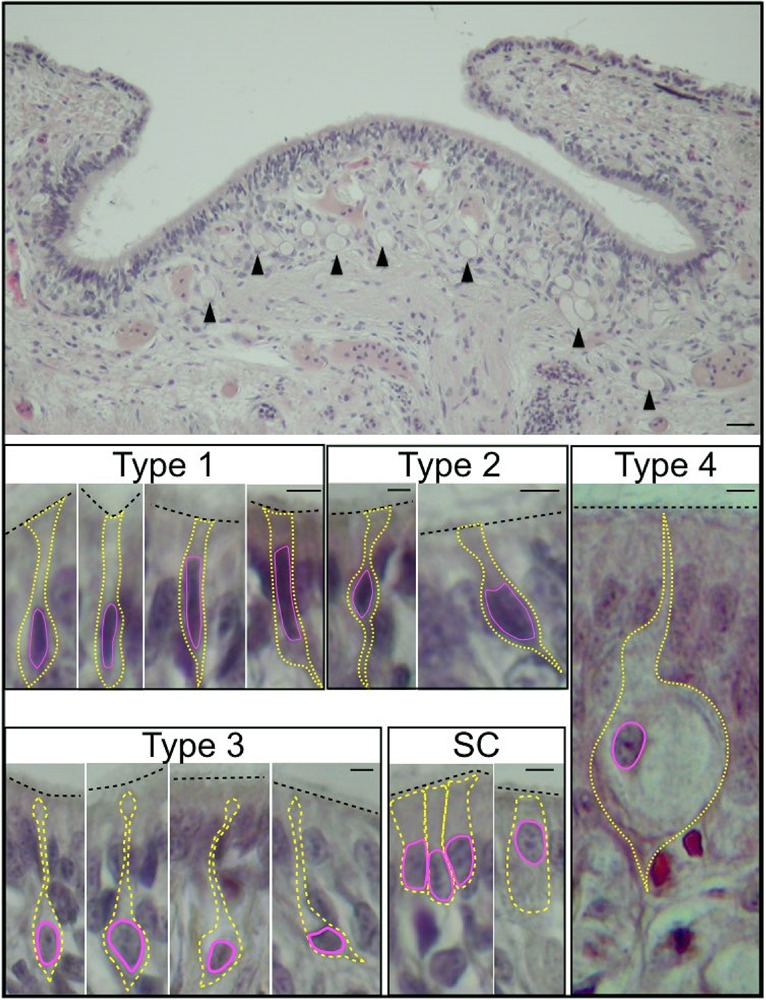
**Transverse section of *O. vulgaris* olfactory organ.** Top: the olfactory epithelium (scale bar=100 µm), arrowheads indicate vacuolated cells (type 4). The sensory cells are shown below: type 1 sensory cells have an elongated nucleus and minimal cytoplasm; type 2 sensory cells have a central nucleus and project to the epithelial surface and to the basal lamina; type 3 sensory cells have a soma occupied by a large nucleus and a long process directed to the surface; type 4 sensory cells are pear shaped cells with a large vacuole, an eccentric nucleus, minimal cytoplasm and a long projection which appears to terminate in cilia. Cylindrical sustentacular cells (SC) emerge onto the epithelial surface with an apical brush border of microvilli. Scale bars=5 µm.

Mayer's haematoxylin/eosin stained slices of the OE revealed five different type of cells, three of them classified as typical olfactory sensory neurons, one with a ring shaped aspect and the last one with a columnar morphology interspersed among the sensory types (Fig. 2).

The first sensory cell type (type 1) has an elongated piriform nucleus, minimal cytoplasm and its apical area is equipped with terminal cilia. Sensory cell type 2 appears characterized by a soma almost totally occupied by the nucleus located in the middle layer of the epithelium, with a broad dendritic process ending with a tuft of cilia on the epithelium surface. The type 3 sensory cells lie deepest within the olfactory epithelium with a large nucleus that fills almost all the soma from which a long dendritic process reaches the epithelial surface with a ciliated terminal.

Under the surface layer of the epithelium, the so called ‘ringed shaped cells’ (Woodhams and Messenger, 1974) (type 4) are characterized by a single large cytoplasmic vacuole, up to 60 µm diameter.

Finally the sustentacular cell type (SC) has a continuous apical brush border of microvilli without cilia. According to previous works on the *O. vulgaris* olfactory organ (Wildenburg, 1997), sustentacular cells are interspersed between OSNs and are jug-shaped with a cytoplasmic process that reaches the epithelial surface. They are characterized by large basal nuclei, occasionally observed in the middle region of the cell (Fig. 2). Still in agreement with Wildenburg (1997) no mucus cells were detected in the *O. vulgaris* OE.

### Measurement of sensory epithelial surface area

The epithelial surface in male and female of *O. vulgaris* is 15±0.5 mm^2^. No significant differences in the structure and morphology of the young male and female olfactory organ were observed.

### 3D reconstruction

Analyzing the histological sections of the two considered postures, the 3D reconstruction of the olfactory organ appears to be radially symmetrical with a mobile central OP surrounded by a fold with raised edges.

**Fig. 3.**
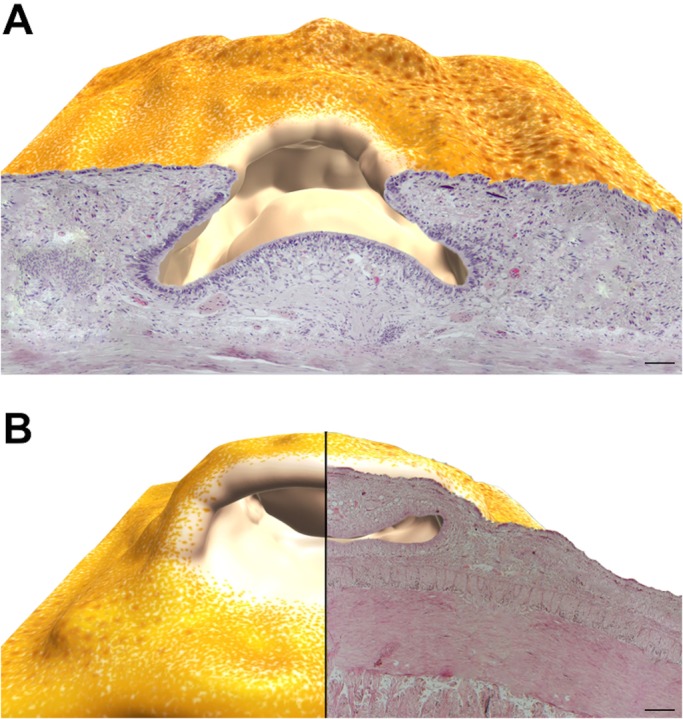
**3D reconstruction of the *O. vulgaris* olfactory organ.** (A) Reconstruction in the relaxed position with the protuberance in the middle of the epithelium surface. (B) Reconstruction of the erect olfactory organ resulting in rotation of the epithelium inside the olfactory pit. Scale bars=100 µm.

Fig. 3 shows the 3D reconstruction resulted from the assemblage of the olfactory epithelium histological sections.

### Proliferating cell nuclear antigen (PCNA) localization

**Fig. 4.**
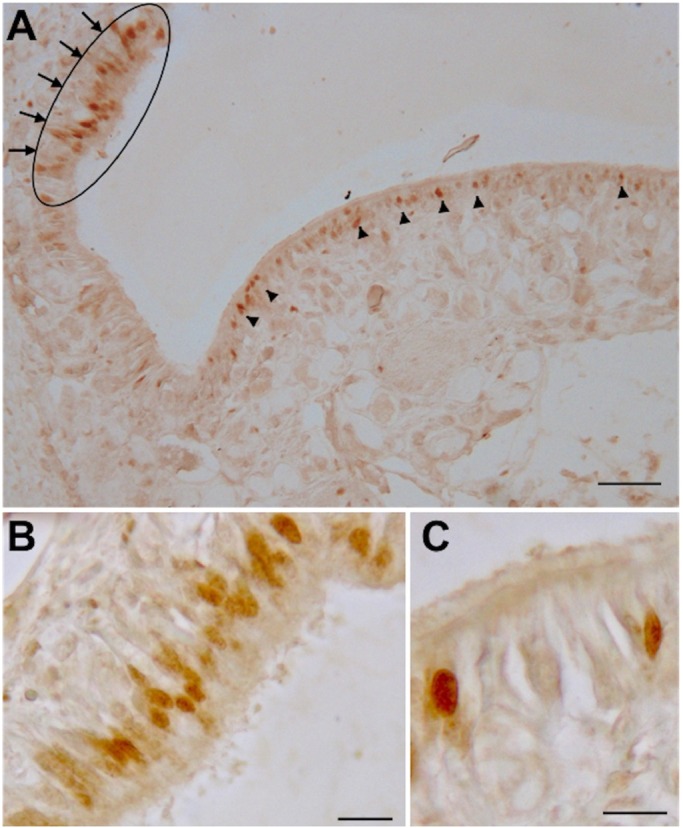
**PCNA immunoreactivity on a transverse section of *O. vulgaris* olfactory organ.** (A) Overview of the olfactory epithelium with several olfactory sensory neurons labeled. The arrowed oval indicates the most proliferative area with a concentration of PCNA immunoreactivity nuclei on the peripheral fold of the epithelium, the arrowheads indicate some scattered PCNA immunoreactivity nuclei on the central epithelium area. (B,C) Magnifications with PCNA immunoreactivity cells in the fold and into the olfactory protuberance, respectively, of the olfactory epithelia. Scale bars=100 µm in A, 10 µm in B,C.

PCNA immunoreactive OSNs appear mainly located on the peripheral folds of the OE, and just a few scattered cells in the central OP area. All the labeled cells are concentrated within the superficial layer of the epithelium. The PCNA immunoreactivity is specifically restricted to the nuclei of type 1 and type 2 OSNs (Fig. 4).

### Olfactory marker protein-like (OMP) localization

**Fig. 5.**
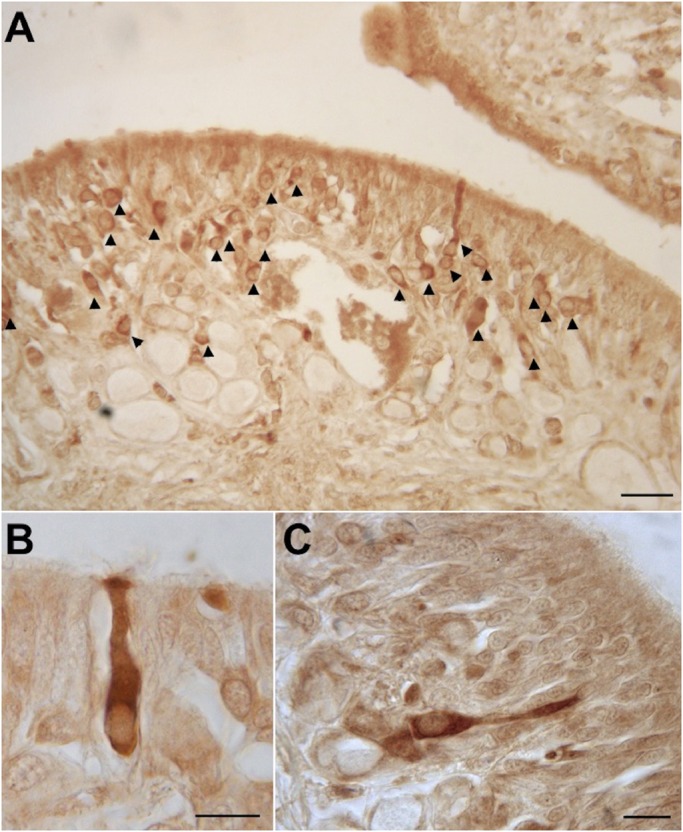
**OMP immunoreactivity on transversal section of *O. vulgaris* olfactory organ.** (A) Overview of the olfactory epithelium, arrowheads indicate many labeled olfactory sensory neurons. (B) Type 2 olfactory sensory neuron OMP immunoreactivity. (C) Type 3 olfactory sensory neuron OMP immunoreactivity. Scale bar=100 µm in A, 10 µm in B,C.

The OMP, which in vertebrates is marker for mature olfactory sensory neurons (Margolis, 1980), is expressed in the cytoplasm, the emerging axon and the dendritic process of types 2 and 3 OSNs only. No OMP immunoreactivity has been observed in types 1 and 4 OSNs. The 3D distribution of OMP immunoreactive OSNs is uniformly scattered in the central OP in which the type 2 cells occupy the upper layer while the type 3 cells occupy the layer below (Fig. 5).

### Characterization of antibodies

#### OMP western blot analysis

**Fig. 6.**
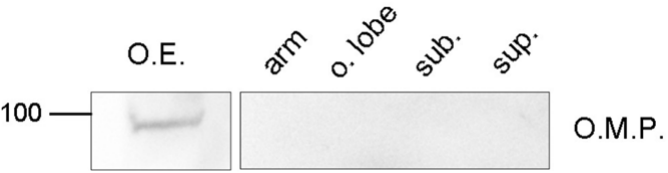
**Western blot analysis of OMP from *O. vulgaris*.** OE: Olfactory epithelium extract showing an immunoreactive band of about 100 kDa. No immunoreactive bands are detected in: arm, optic lobe (o. lobe), subesophageal mass (sub.) and supraesophageal mass (sup.).

On SDS-PAGE of membrane proteins from *O. vulgaris* OE the antibody revealed a distinct OMP immunopositive protein at ∼100 kDa (Fig. 6). *O. vulgaris* arm, optic lobe, subesophageal mass and supraesophageal mass extracts treated with the same antibody showed a negative result (Fig. 6).

#### Dot blot assay

A dot blot assay for anti-OMP showed positive immunoreactivity with protein extract from *O. vulgaris* OE, but negative immunoreactivity with protein extract from supra- and suboesophageal masses, as well as from optic lobe and arm. Negative immunoreactivity was observed when protein extract from OE was incubated with pre-absorbed antibody (Fig. S1).

#### Sequence alignment

Alignment of mouse PCNA whole protein sequence with PCNA protein sequence annotated in *Octopus bimaculoides* genome (Albertin et al., 2015), showed an identity of 77% (Fig. S2).

## DISCUSSION

In this study we provide an unprecedented view of the olfactory epithelium of *O. vulgaris* in term of anatomy and turnover capabilities. We describe the detailed morphology of young male and female octopus olfactory epithelium, and using a combination of classical morphology and 3D reconstruction techniques we propose a new classification for *O. vulgaris* OSNs. Furthermore using specific markers such as OMP and PCNA we has been able to identify and differentially localize, both mature olfactory sensory neurons (OMP immunoreactivity) and olfactory sensory neurons involved in epithelium turnover (PCNA immunoreactivity).

### Histology and cell types

We recognize a pseudo-stratified, columnar and ciliated epithelium that revealed the presence of four OSNs and just one sustentacular cell type.

We partially agree with previous descriptions of olfactory cell types observed respectively in the decapodiformes *Lollingula brevis* (Emery, 1975), *Loligo vulgaris* (Wildenburg and Fioroni, 1989) and *Sepia officinalis* (Wildenburg, 1990), and octopodiformes, adult *O. vulgaris* (Woodhams and Messenger, 1974), *Octopus joubini* (Emery, 1976) and post-hatching *O. vulgaris* and *Eledone moschata* (Wildenburg, 1997).

We found just one epithelial cell non-sensory type (SC) in contrast with what was found in decapodiformes and some octopodiformes in which two epithelial non-sensory cells have been described. However our SC type corresponds to epithelial cell type 1 described in decapodiformes and octopodiformes by Wildenburg (1990, 1997) (Table 1).

**Table 1.**
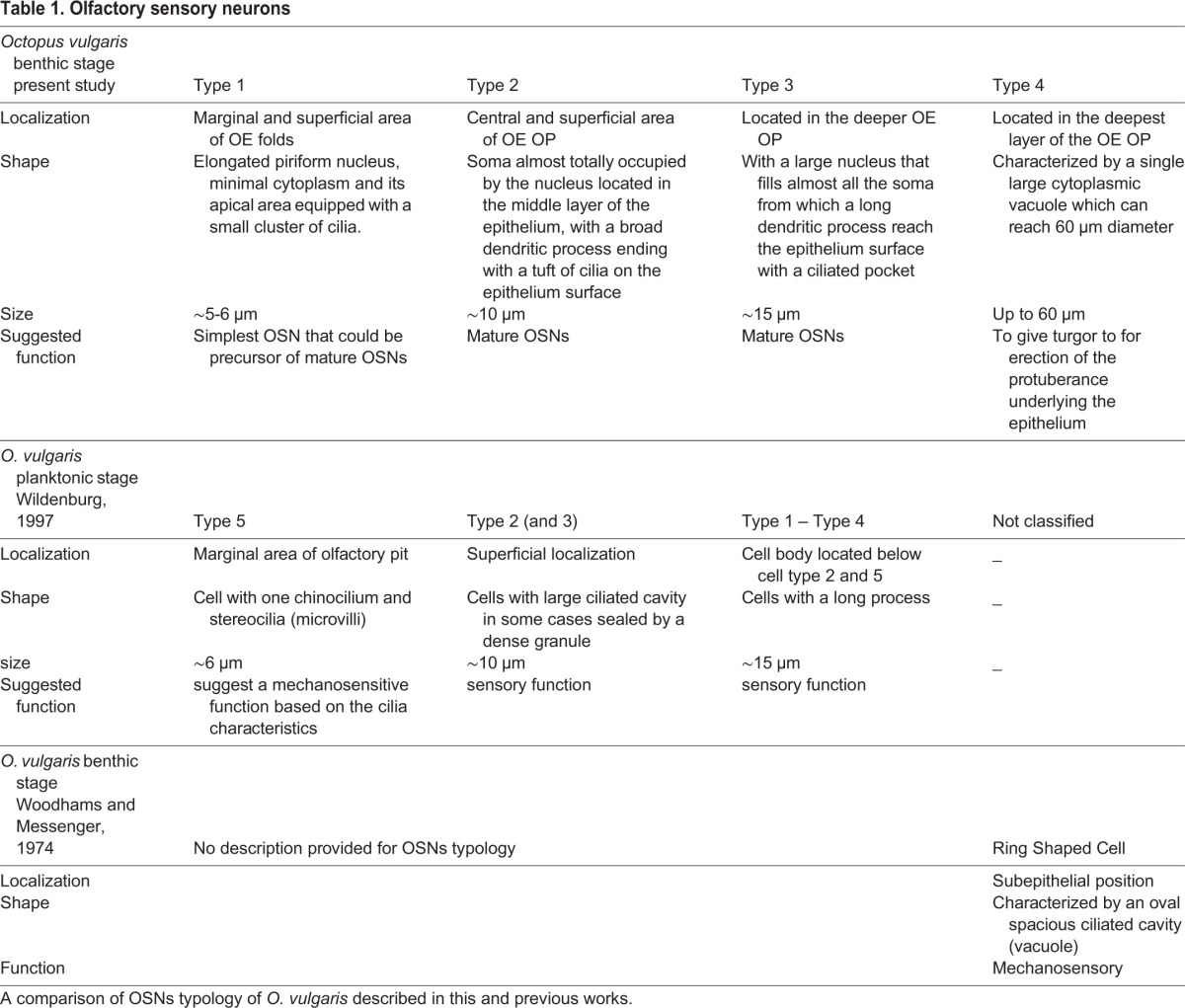
**Olfactory sensory neurons**

Of the four OSN types we discovered, the type 3 in our classification corresponds to type 1 described by Wildenburg (1990, 1997), while our type 2 corresponds to type 2 and its variations (Table 1). The types 1 and 4 in our classification appear characteristic of *O. vulgaris* and they correspond respectively to type 5 described by Wildenburg (1997) and to ring shaped cells described by Woodhams and Messenger (1974) (Table 1).

Differently from what was described in previous works on decapodiphormes and in agreement with Wildenburg (1997), we have observed that both cell types 3 and 4 are located in the deep OE forming a layer under the epithelium surface (Table 1). Interestingly the cell type 5 that Wildenburg (1997) defined characteristic of *O.vulgaris* planktonic stage only, occurs in our preparations classified as type 1 in both male and female young benthic *O.vulgaris* OE (Table 1).

Our cell type 1 in the benthic stage (cell type 5 planktonic stage; Wildenburg, 1997) represents the simplest OSNs letting us speculate that all the OSNs could possibly have evolved from them according to our PCNA immunoreactivity results in which the majority of the immunoreactive cells are type 1. This contrasts with the hypothesis advanced by Wildenburg (1997) witch the type 4 cells are the precursor of all the others.

The sensory cells evolve and differentiate in more complex forms (Graziadei, 1965; Boyle, 1986) in which the cells that lack a pore leading to the external environment were previously considered as either immature chemoreceptors or as mechanoreceptors (Wildenburg and Fioroni, 1989; Wildenburg, 1997). In our view the cells that lack a pore leading to the external environment represent a more derived and specialized form that may originate from type 1 in our classification (Table 1).

The type 4 cells, classified also as ‘ring shaped’ cells (Woodhams and Messenger, 1974), deserve particular attention given that their shape is quite specific. Besides their uncommon size, they possess a large vacuole and are mainly distributed in the deeper layer of the central part of the OE where they appear to give turgor to the OP that characterizes the olfactory organ shape. Previous electron moicroscopic studies on *O. vulgaris* and other species of cephalopods, revealed the presence of cilia in the vacuole. For their shape and position in the OE some authors (Emery, 1976; Woodhams and Messenger, 1974) hypothesized that the type 4 cells could work as mechanosensory cell type suggesting a double function of the olfactory organ (Table 1). However, we suggest that the function of this cell type is related to the architecture and structural plasticity of the whole organ (see 3D reconstruction) and thus determines whether it is relaxed or erect but this hypothesis remains to be experimentally tested.

### 3D reconstruction and structural plasticity

The 3D reconstruction provides a useful tool to understand the spatial configuration of the olfactory organ. Following the virtual representation obtained by reassembling the histological sections from what appear to be relaxed and erect postures of the organ we argue that it has an intrinsic capability of movement that allows the animal to orientate it to detect the spatial gradient of chemical cues. This could help their navigation and spatial memory abilities (Huffard, 2013). However, touch and olfaction are a part of a multimodal system of information transfer. The synchronous use and integration of different signals using different channels (touch and olfaction) have the advantage to improve recognition, discrimination and memory of inputs by the environment (Partan and Marler, 2005).

The olfactory organ in cephalopods has been described often as a pit or an OP. Wildenburg (1990) even hypothesized an adaptive evolution of the organ related to different hatchling types of different species: a pit shaped organ in bottom living hatchlings, and a bulging organ in pelagic hatchlings such as *O. vulgaris*. Based on our 3D reconstruction we define the *O. vulgaris* olfactory organ as a pit with an erectile internal OP.

### Epithelial proliferation

#### Proliferating cell nuclear antigen (PCNA) localization

Olfactory sensory cells in all vertebrates are characterized by cycles of birth, maturation, and death (Graziadei and Monti Graziadei, 1978). This proliferation is remarkable given that the olfactory receptor cells are neurons, cells that are not generally considered to undergo neurogenesis in adults. The same labeling technique used to document turnover in vertebrates shows that OSNs in the anterior tentacles (olfactory organs) of snails also turn over (Chase and Rieling, 1986). Functional constancy in diverse groups of animals argues that turnover is a common adaptive property of OSNs. We verify the presence of OSNs proliferation in *O. vulgaris* based on the presence and distribution of PCNA immunoreactivity.

PCNA is a nuclear protein synthesized in the G1 and S phases of the cell cycle and, therefore, correlated with the cell proliferative stage (Jaskulski et al., 1988; Tsurimoto, 1999; Wullimann and Puelles, 1999; Rankin et al., 2004), thus represents a valuable marker of cell proliferation (Derenzini et al., 1990, 1995; Öfner et al., 1992). PCNA has been recently localized in octopus arm regeneration process (Fossati et al., 2013).

We have observed that PCNA immunoreactive OSNs are mainly located in the external layer of the olfactory epithelium lateral folds, with sporadic immunoreactive OSNs observed on the central OP. This observation suggests a migratiory wave from the top/lateral layer of the epithelium to the central/deeper one. No positive cells were found in deeper layers. Since the majority of PCNA immunoreactive cells are type 1, and they are mainly located in the marginal region of the OE, we suggest that the epithelial proliferation starts from the periphery of the organ up to the center. It is also intriguing to observe that the type 1 cells do not have any sign of further sensory specializations such as the presence of a ciliated pore or internal vacuole (Fig. 2), implying that this type of cell may be a precursor of all the OSNs in contrast to Wildenburg (1997) when he described cell type 4 (ring shaped cells) as the ‘ontogenetic stage of other sensory cell types’. Cell type 4 has never been observed to be positive to the PCNA antibody.

### Chemosensory function

#### Olfactory marker protein-like (OMP) localization

The OMP is a highly abundant small cytoplasmic protein whose gene expression is highly restricted to mature olfactory chemosensory neurons and is phylogenetically conserved among vertebrates (Margolis, 1980; Danciger et al., 1989; Reisert et al., 2007).

In invertebrates an olfactory sensory neuron-specific protein has been cloned in the mollusk land snail *Eobania vermiculata* (Mazzatenta et al., 2004).

The OMP immunoreactivity has been here detected for the first time in the olfactory organ of invertebrate OSNs suggesting an even more conserved function of this protein. The presence of OMP immunoreactive OSNs in *O. vulgaris* supports the chemosensory function of the ‘so called olfactory organ’ (Woodhams and Messenger, 1974).

The OMP immunoreactivity results are mainly confined to the cytoplasm, including the emerging axon and the dendritic process, of types 2 and 3 OSNs. This selective localization strongly supports the hypothesis that these cell types are the mature form of OSNs deriving from the type 1 cells.

The absence of OMP immunoreactivity in the lateral side of the organ suggests a turnover of this epithelium with mature OSNs located in the medial OP. Furthermore the fact that cell type 4 never shows OMP immunoreactivity strongly suggests that the ‘ring shaped cells’ do not have an olfactory function. Moreover, both controls of anti-OMP specificity, western and dot blot analyses clearly showed that immunoreactivity is restricted to just the OE protein extract strongly supporting its conserved function. The protein that cross reacts with anti-OMP has a molecular weight of about 100 kDa, and that homologue and heterologue pre-absorption tests performed with proteins extracts from *O. vulgaris* OE, as well as recombinant rat OMP, abolished completely OMP immunoreactive band in western and dot blot analysis. This biochemical data further support the contention that the immunoreactivity reported here is due to the presence of an OMP isoform.

Our previous finding about the presence of neuropeptides involved in the regulation of food intake and reproduction (Di Cosmo and Polese, 2014; Polese et al., 2015) mainly localized in cell type 2 and 3, together with the data presented in this study where the OMP immunoreactivity is confined to these two types of OSNs, strongly suggests that OSNs change their position and role as they mature.

**Fig. 7.**
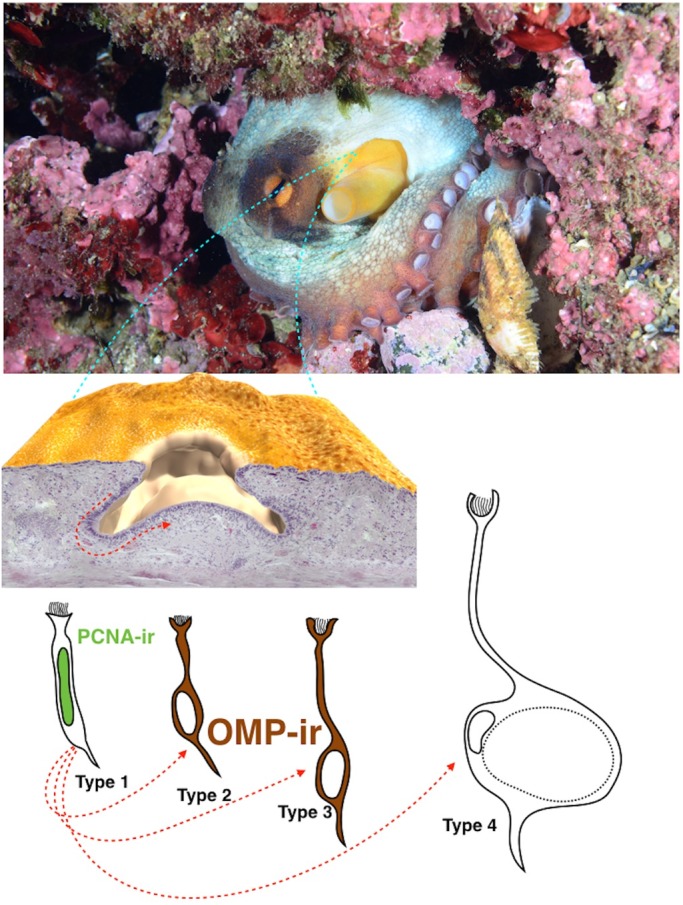
**Overview of the *O. vulgaris* olfactory organ.** Starting from the top, showing the anatomical position of the organ, followed by its 3D reconstruction. Below, the different type of OSNs are represented. The red arrows indicate the pathway of maturation and differentiation of OSNs.

In conclusion this work represents the first attempt to characterize the olfactory organ of *O. vulgaris* (summarized in Fig. 7) opening new perspectives about the role-played by the olfaction in the complex behavioral patterns shown by this fascinating animal. In the near future we will try to confirm the functional role of the olfactory organ performing behavioral experiments.

## MATERIALS AND METHODS

### Animals, dissection, and fixation

Specimens of young *O. vulgaris* males and females (*n*=12, 6 males and 6 females, weight, ±400 g) were collected from Bay of Naples and maintained in aquarium tanks under the same conditions as reported in Fiorito et al. (2014) and Polese et al. (2014).

Our research conformed to European Directive 2010/63 EU L276, the Italian DL. 4/03/2014,n. 26 and the ethical principles of Reduction, Refinement and Replacement (protocol n. 0124283-08/11/2012).

Animals were anesthetized by isoflurane vaporized into the bathing medium as reported previously (Polese et al., 2014) and the olfactory organs were dissected in sterile conditions.

The tissues were fixed in Bouin's solution for 24 h at room temperature, then washed and dehydrated in ethanol, cleared in Bioclear (Bio-WORLD), and embedded in paraffin.

### Histology protocol

Transverse sections (7 µm) were cut on microtome and mounted on albumin-coated slides, cleared, rehydrated and processed for routine Mayer's hematoxylin and eosin staining.

### 3D reconstruction

Two different positions of the olfactory organ were considered for 3D reconstructions. The first is assumed to be the relaxed position when the organ seats at the base of the pit. The second position is when the organ is erected out of the pit (Fig. 1).

The 3D reconstruction of the *O. vulgaris* olfactory organ results from the overlapping of 370 serial histological sections of 7 µm thick and stained with haematoxylin/eosin, obtained from two olfactory organs fixed in the two considered postures. Pictures of each serial histological section were taken using a Leica DM-RB microscope equipped with Canon power shot S50 digital camera. All the pictures taken were assembled and analyzed (Blender and 3D Coat software).

### Basic immunohistochemical protocol

We used methods based on those reported previously for studying the nervous system of *O. vulgaris* (Di Cosmo and Di Cristo, 1998) and *Sepia officinalis* (Di Cosmo et al., 2004). Transverse sections of olfactory organs from both sexes were cleared, rehydrated, washed in phosphate saline buffer (PBS) and treated for immunohistochemical analyses.

After incubation with both primary (Table 1) and biotinylated secondary antibodies, and several rinses in PBS, streptavidin conjugated to horseradish peroxidase (dilution 1:200, from Life Technologies Carlsbad, CA, USA) was placed on the sections for 1 h. Then 3% DAB (3.30-diaminobenzidine tetrahydrochloride; Sigma Aldrich, St. Louis, MO, USA) with 0.03% hydrogen peroxide (Sigma Aldrich) in Tris buffer (0.05 M, pH 7.6) was used as chromogen and slides were dehydrated and mounted in Permount (Thermo Fisher Scientific, Waltham, MA, USA).

### Anti-proliferating cell nuclear antigen immunohistochemistry (anti-PCNA)

Anti-PCNA antibody was used as a molecular marker of cell cycle progression and DNA replication. Proliferating cell nuclear antigen was detected using monoclonal mouse anti PCNA (dilution 1:10,000; Sigma Aldrich; #P8825 RRID: AB_477413). Sections were incubated for 20 min with 1% normal horse serum (Life Technologies) and then rinsed in anti-PCNA at 4°C overnight in humid chamber. The sections after many washes in PBS were incubated with horse anti-mouse secondary antibody biotin conjugated (dilution 1:200; Thermo Fisher Scientific) for 1 h at room temperature.

### Anti-olfactory marker protein immunohistochemistry (anti-OMP)

Anti-OMP was used as a molecular marker of mature olfactory chemosensory neurons. Olfactory marker protein was detected using polyclonal goat anti-OMP (dilution 1:10,000; Wako, Richmond, VA, USA; #019-2229 RRID: AB_664696). After incubation for 20 min with 1% normal rabbit serum (Life Technologies), sections were rinsed in anti-OMP at 4°C, overnight, in a humid chamber. After many washes in PBS the sections were incubated in rabbit anti-goat biotin-conjugated secondary antibody (dilution 1:200; Thermo Fisher Scientific) for 1 h at room temperature.

### Characterization of antibodies

Anti-OMP specificity has been supported by loss of labeling in controls treated with antibody pre-absorbed with the antigen at 5 µM final concentration (recombinant rat OMP kindly provided by F. Margolis, University of Maryland School of Medicine, USA). Given that an OMP has not been annotated in *O. bimaculoides* genome, specificity was tested with a western blot and dot blot assay. Specificity of both secondary antibodies has been tested with omission of primary antibody.

#### Western blot analysis

Total proteins were extracted from homogenate of olfactory epithelium (*n*=8), as well as from arm, optic lobe, sub-supraoesophageal masses, and quantified by Bradford Protein Assay, using a BSA standard, according to manufacturer's instructions (Bio-Rad Laboratories, Inc., Hercules, CA). After 10% sodium dodecylsulphate (SDS)-polyacrylamide gel electrophoresis, proteins were transferred on nitrocellulose membrane (Whatman) and incubated for 30 min in a blocking solution (non-fat milk 5% in PBS). Membranes were incubated in antibody solution (1:1000 anti-OMP in non-fat milk 5%) at 4°C overnight. After several rinses with PBS-T (PBS with 0.1% of Tween 20), membranes were incubated with secondary antibodies (1:5000) for 1 h at room temperature. Immunopositive band was visualized using the SuperSignal West Pico Chemiluminescent Substrate in accordance with the manufacturer's instructions (Pierce Biotechnology, Inc., Rockford, IL, USA) using a Chemidoc EQ System (Bio-Rad).

#### Dot blot assay

2 µl (200 ng/μl) of total protein extract from *O. vulgaris* tissues respectively: olfactory epithelium (OE), supra- and suboesophageal masses, optic lobes and arm were applied on nitrocellulose membrane (Whatman) and let dry at room temperature. After 1 h incubation with non-fat milk 5% blocking solution, we incubated with anti-OMP (dilution 1:500; Wako; #019-2229 RRID: AB_664696) overnight at 4°C. The membrane was then washed extensively using Tris buffer with Tween 20 (Sigma Aldrich) 0.05% (TBS-T) and subsequently incubated with a rabbit anti goat horseradish peroxidase conjugated (dilution 1:5000; Thermo Fisher Scientific) for 1 h. After several rinses with TBS-T, immunopositive dots were visualized using the SuperSignal West Pico Chemiluminescent Substrate (Pierce Biotechnology) in accordance with the manufacturer's instructions using a Chemidoc EQ System (Bio-Rad).

Anti-PCNA specificity has been determined in a recent study on octopus arm regeneration (Fossati et al., 2013) and supported by loss of labelling in controls with the antibody pre-adsorbed with its antigen. Furthermore, using CLUSTALW2 database, we performed an alignment of mouse PCNA whole protein sequence with PCNA protein sequence annotated in *Octopus bimaculoides* genome recently published (Albertin et al., 2015).

### Data imaging

Images were obtained as described above and were processed using Photoshop CS2 (Adobe Systems, San Jose, CA). Further processing was restricted to image-wide intensity and contrast adjustment. Schematics and multi-panel figures were created, assembled and labeled in Keynote (Apple Inc. Cupertino, CA, USA).

### Measurement of sensory epithelial surface area

The epithelium surface areas were calculated measuring the external margin of the epithelium in the most central section of the processed organs. Since the OE is circular, its area was calculated, after linearizing the epithelial margin, using the formula for the area of the circle (πr^2^).
